# MicroRNA‐17 as a promising diagnostic biomarker of gastric cancer: An investigation combining TCGA, GEO, meta‐analysis, and bioinformatics

**DOI:** 10.1002/2211-5463.12496

**Published:** 2018-08-30

**Authors:** GaoFeng Hu, QianWen Lv, JiaXiu Yan, LiJun Chen, Juan Du, Ke Zhao, Wei Xu

**Affiliations:** ^1^ Department of Clinical Laboratory The First Hospital of Jilin University Changchun China; ^2^ Department of Neonatology The First Hospital of Jilin University Changchun China; ^3^ Institute of Virology and AIDS Research The First Hospital of Jilin University Changchun China

**Keywords:** biomarker, gastric cancer, gene expression omnibus, meta‐analysis, microRNA‐17, TCGA

## Abstract

Integrated studies of accumulated data can be performed to obtain more reliable information and more feasible measures for investigating potential diagnostic biomarkers of gastric cancer (GC) and to explore related molecular mechanisms. This study aimed to identify microRNAs involved in GC by integrating data from The Cancer Genome Atlas (TCGA) and Gene Expression Omnibus. Through our analysis, we identified hsa‐miR‐17 (miR‐17) as a suitable candidate. We performed a meta‐analysis of published studies and analyzed clinical data from TCGA to evaluate the clinical significance and diagnostic value of miR‐17 in GC. miR‐17 was found to be upregulated in GC tissues and exhibited a favorable value in diagnosing GC. In addition, we predicted that 288 target genes of miR‐17 participate in GC‐related pathways. Enrichment of Kyoto Encyclopedia of Genes and Genomes pathway, Gene Ontology analysis, and protein–protein interaction analysis of the 288 target genes of miR‐17 were also performed. Through this study, we identified possible core pathways and genes that may play an important role in GC. The possible core pathways include the cAMP, phosphoinositide‐3‐kinase–Akt, Rap1, and mitogen‐activated protein kinase signaling pathways. miR‐17 may be involved in several biological processes, including DNA template transcription, the regulation of transcription from RNA polymerase II promoters, and cell adhesion. In addition, cellular components (such as cytoplasm and plasma membrane) and molecular functions (such as protein binding and metal ion binding) also seemed to be regulated by miR‐17.

**Abbreviations**
AUC: area under the curve
DAVID: database for annotation, visualization, and integrated discovery
DEM: differentially expressed miRNA
GC: gastric cancer
GEO: gene expression omnibus
GO: Gene Ontology
KEGG: Kyoto Encyclopedia of Genes and Genomes
miR‐17: hsa‐miR‐17
miRNA: microRNA
PPI: protein–protein interaction
ROC: receiver operating characteristic
RPM: reads per million
SMD: standard mean difference
SNR: signal‐to‐noise ratio
SROC: summary receiver operating characteristic
TCGA: The Cancer Genome Atlas

Gastric cancer (GC) ranks as the second most frequent cancer and is the second leading cause of cancer‐related death, following lung and bronchus cancer, in China 1. The definite clinical diagnosis depends on upper‐abdominal endoscopy with biopsy and cytological examination, with a diagnostic accuracy of approximately 95–99% for both types of GC 2. Nevertheless, limited methods exist for screening, early diagnosis, and recurrence monitoring with reduced discomfort and increased detection rate. Even the most prevalent blood biomarkers for gastrointestinal tumors, such as CEA, CA19‐9, and CA72‐4, often disappoint clinicians and GC patients because their positive detection rate is no more than 41% 3. It is well known that GC tumorigenesis, progression, and metastasis are highly related to dysregulated gene expression. Thus, identifying genes that are differentially expressed at the DNA and RNA levels between tumor and normal tissues may benefit the diagnosis, prognosis, and prediction of GC and help elucidate the molecular mechanisms underlying oncogenesis and therapeutic strategies 4, 5.

MicroRNAs (miRNAs) are small, noncoding RNAs that are 21–24 nucleotides in length and participate in post‐transcriptional regulation by binding to the 3′ untranslated regions of target genes, along with 5′ untranslated regions and coding sequences, leading to translational inhibition and cytoplasmic degradation 6, 7, 8. The aberrant expression of miRNAs is involved in multiple diseases, including various types of cancers. With advancements in research, miRNA‐mediated regulatory networks such as miRNAs–lncRNA–mRNA, miRNAs–circRNA–mRNA, and miRNA–mRNA–miRNA have been gradually used to elucidate the complicated molecular mechanisms underlying tumorigenesis, disease progression, invasion, and metastasis 9, 10, 11, 12, 13. In the past few years, studies in many fields have focused on the utilization of miRNAs in GC, ranging from diagnosis to therapies 14, 15. Accumulated knowledge has provided abundant resources for integrated studies to obtain more reliable information and more feasible measures in the field of medicine. In recent years, microarrays and sequencing technology have been extensively used as efficient tools for the identification of differentially expressed miRNAs (DEMs). Some widely available open access databases include The Cancer Genome Atlas (TCGA) and the Gene Expression Omnibus (GEO). Through searching the TCGA and GEO databases, we identified hsa‐miR‐17 (miR‐17) as a promising candidate for the diagnosis of GC and explored the associated molecular mechanism. Hence, this study aimed to comprehensively investigate the clinical significance and diagnostic value of miR‐17 in GC via meta‐analysis based on the two databases, the literature, and bioinformatics analysis.

## Materials and methods

### miRNA‐seq data from TCGA database

Publicly available miRNA‐seq data on miRNA levels in GC samples were directly downloaded from TCGA data portal (http://cancergenome.nih.gov/) with file filters [Transcriptome Profiling (Data Category), miRNA Expression Quantification (Data Type), miRNA‐Seq (Experimental Strategy)], and case filters [TCGA‐STAD (Project)] on 12 December 2017. The corresponding clinical data were downloaded using Xena (http://xena.ucsc.edu/) from TCGA database. There were 491 files with a total of 436 stomach adenocarcinoma (STAD) samples. Furthermore, 41 cases had miRNA‐seq data from matched adjacent normal gastric mucosal tissues, whereas another 24 cases lacked pathological staging information. Finally, the miRNA‐seq data for 412 GC samples and 41 normal stomach mucosal samples were obtained for further analysis. Reads per million (RPM) values were extracted for 1882 mapped miRNAs in each sample. In addition, we cleaned the data using Python. DEMs between GC samples with pathological stages I–IV and normal stomach control samples were identified by calculating the fold change (FC) (|log_2_(FC)| > 1 and *P* < 0.05) with the r package deseq. One‐way analysis of variance or Student's *t*‐test was used to analyze the relationship between the relative miRNA expression levels and clinical characteristics with spss statistics version 20.0 (IBM Corp., Armonk, NY, USA). *P* < 0.05 was considered statistically significant.

### Microarray profiles from the GEO database

Microarray profiles (up to 11 January 2018) related to GC were obtained from the GEO database (http://www.ncbi.nlm.nih.gov/geo/) with the following search strategy: (miR OR miRNA OR microRNA) AND (malignant OR tumor OR tumour OR cancer OR carcinoma OR neoplasm OR neoplasms) AND (gastric OR stomach). The microarrays that met the following criteria were collected: (a) studies including at least 20 samples and (b) examination of miRNA expression in tissues, serum, or blood samples of GC patients. Microarrays that did not provide useful data for analysis were excluded. Finally, 12 GEO datasets, namely GSE93415, GSE78775, GSE63121, GSE54397, GSE26595, GSE33743, GSE30070, GSE28770, GSE85589, GSE59856, GSE61741, and GSE31568, were included in the present study. The DEMs between GC and healthy control samples in each GEO dataset were ranked according to the signal‐to‐noise ratio (SNR) using morpheus (http://software.broadinstitute.org/morpheus/), an online web tool. The top 250 DEMs in each direction were chosen for further analysis. An independent Student's *t*‐test or a paired *t*‐test was performed to calculate the difference in the levels of a particular miRNA between GC and healthy control samples. *P* < 0.05 was considered statistically significant.

### Real‐time PCR data of miR‐17‐5p from published studies

A literature search (up to 11 January 2018) was conducted using the following databases: PubMed, Web of Science, EMBASE, and Cochrane. The following search strategy was used: (miR‐17 OR miRNA‐17 OR microRNA‐17 OR miR17 OR miRNA17 OR microRNA17 OR miR 17 OR mirna 17 OR microrna 17) AND (malignant OR cancer OR tumor OR tumour OR neoplasm OR carcinoma OR neoplasms) AND (gastric OR stomach). Eligible studies met the following criteria: (a) the studies were original articles; (b) the studies were on human GC patients; (c) miR‐17‐5p (or miR‐17) expression in GC tissues, serum, or plasma was measured by qRT‐PCR; (d) the required data could be determined or extracted from the original articles; (e) studies with the largest patient sample size were included if the data were published in multiple papers; and (f) the studies were published in English. Studies were excluded if they were published as an abstract, summary, case report, comment letter, review, or editorial. The assessment and selection of eligible studies were performed independently by two authors (G‐FH and Q‐WL). Controversial studies were reassessed by a third author (J‐XY) for consensus, and agreements were reached by discussion. After being carefully reviewed, the required data were extracted from the included studies using getdata graph digitizer version 2.26 (Germany).

### Meta‐analysis

The following data were extracted from each included study for meta‐analysis: sample number and the mean ± SD of the GC group and healthy control group, true positivity, false positivity, false negativity, and true negativity. stata 12.0 (StataCorp, College Station, TX, USA) was used to conduct the meta‐analysis. The ‘metan’ module of stata 12.0 was used to determine the standardized mean differences (SMDs) and 95% confidence intervals (CIs) for pooled values. Heterogeneity was evaluated using Cochran's *Q* (chi‐square test) and the *I* ^2^ test. *P* < 0.1 for the *Q* test and/or *I* ^2^ > 50% were considered to indicate significant heterogeneity. If no obvious heterogeneity was detected, a fixed‐effects model was used. Otherwise, a random‐effects model was used. Additionally, subgroup analyses were performed based on the features of different studies to identify the source of heterogeneity. Publication bias was detected using Deeks's funnel plot asymmetry test. *P* ≥ 0.05 was considered to indicate the lack of publication bias. The summary receiver operating characteristic (SROC) curve was constructed according to the sensitivity and specificity.

### Prediction of miRNA target genes

The prediction of possible miRNA target genes was performed using the following databases: miRTarbase 16, Tarbase v.8 17, Targetscan7.1 18, microT‐CDS 19, RNA22 2.0 20, PicTar‐vet 21, miRDB 22, PolymiRTs 23, miRSystem 24, Targetminer (http://www.isical.ac.in/~bioinfo_miu/targetminer20.htm), miRecord (http://c1.accurascience.com/miRecords/), and miRWalk3.0 (http://129.206.7.150/). Genes overlapping in more than five databases were selected. It should be noted that target genes gained from Tarbase, mirTarBase, miRWalk3.0, and miRecord were experimentally validated. In addition, we identified differently expressed genes related to the pathological stage of GC using LinkedOmic 25, an online web tool based on TCGA, and collected the genes with *P* < 0.05. Finally, the intersection of miRNA‐related genes and GC‐related ones was included for the bioinformatics analysis.

### Integrative bioinformatics analysis

The achieved target genes of miRNAs were pooled for Kyoto Encyclopedia of Genes and Genomes (KEGG) pathway analysis and Gene Ontology (GO) enrichment using Database for Annotation, Visualization and Integrated Discovery (DAVID) (https://david.ncifcrf.gov/). Protein–protein interaction (PPI) networks were constructed using string version 10.5 (http://string-db.org/cgi/input.pl).

## Results

### miR‐17 at the intersection of the TCGA and GEO datasets for GC

**Figure 1:**
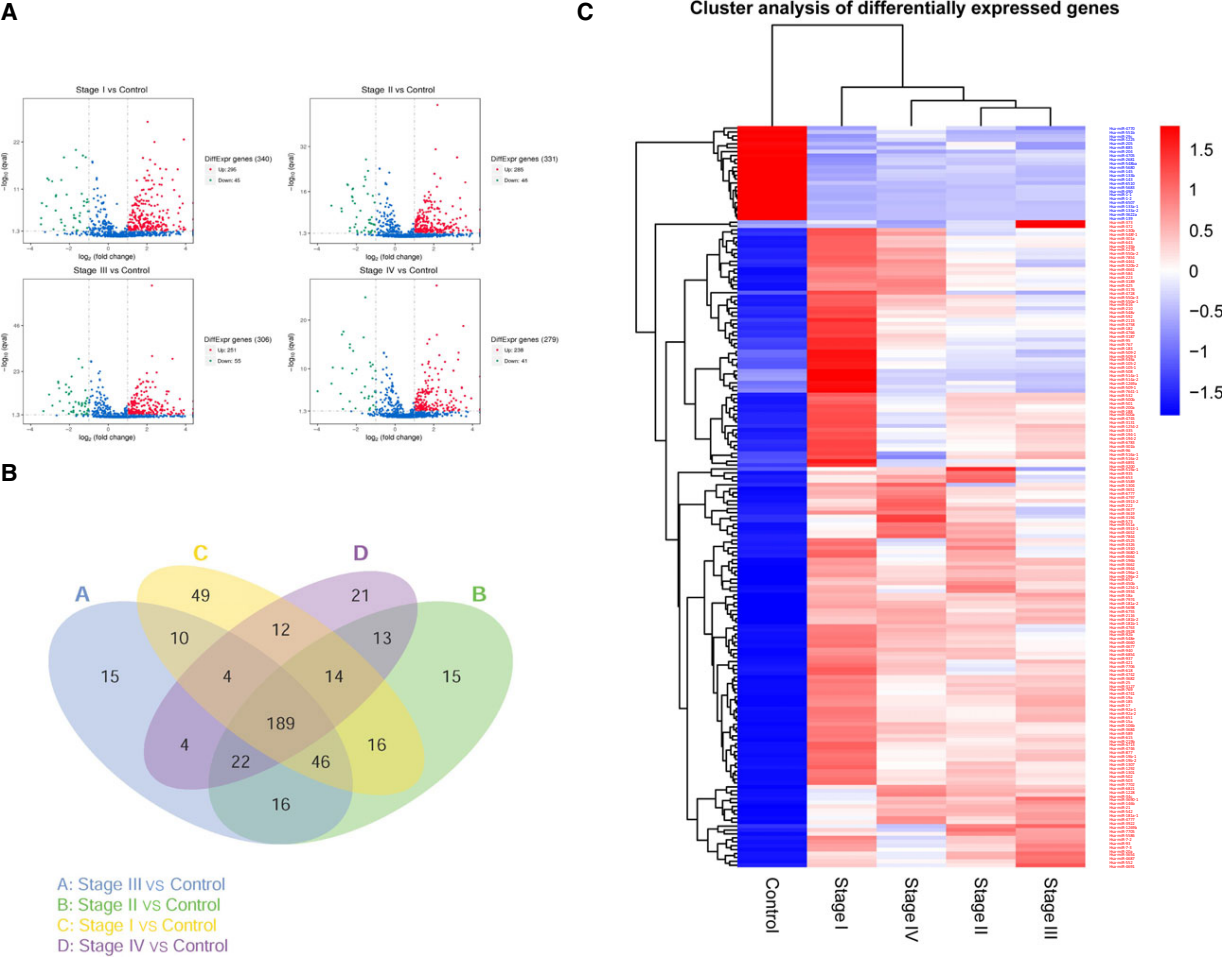
Identification of DEMs in GC based on TCGA database. (A) Volcano plots between healthy controls and the stage I–IV groups in TCGA database. (qval = *P* value) (B) The corresponding Venn diagram of 189 DEMs. (C) The heat map of 189 DEMs, including 165 upregulated miRNAs and 24 downregulated miRNAs.

Data for a total of 412 GC patients (266 males and 146 females) and 41 healthy control individuals were obtained from TCGA datasets, and RPM values of 1882 mapped miRNAs of each subject were extracted. The GC patients were divided into the following four groups, according to the pathologic stage: stage I (*n* = 58), stage II (*n* = 128), stage III (*n* = 183), and stage IV (*n* = 43). We compared the RPM data of 1882 miRNAs between the five groups. In the beginning, we attempted to identify miRNAs that were differentially expressed in all five groups. However, this strategy was not successful, as the intersection of DEMs was zero. Hence, we separately identified the DEMs between the healthy control and stage I–IV groups. The relevant volcano plots are shown in Fig. 1A. The four groups had 189 DEMs in common, including 165 upregulated miRNAs and 24 downregulated miRNAs identified from TCGA database. The corresponding Venn diagram and heat map of 189 DEMs are shown in Fig. 1B,C.

**Figure 2:**
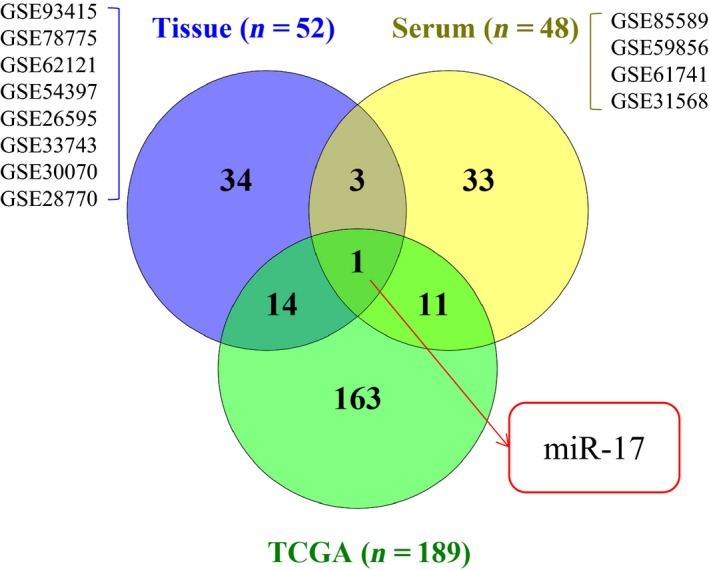
Identification of DEMs. miR‐17 was at the intersection of TCGA and GEO datasets for GC.

We searched the GEO database, and a total of 12 eligible GSE microarrays were included in the present study (Table 1). Because of the differences in sample types of GSE microarrays, the common DEMs were separately examined. Notably, we ignored the differences between the 3p and 5p arms of miRNAs when identifying the DEMs in GSE datasets on account of the names of miRNAs in the TCGA database. miR‐17 was the only miRNA appearing in all of the GSE datasets and the TCGA database. The corresponding Venn diagram is shown in Fig. 2. The 5p and 3p arms of miR‐17 among the top 500 miRNAs in GSE datasets according to the calculated SNR using morpheus are shown in Table 2; miR‐17‐5p and miR‐17‐3p are also known as miR‐17 and miR‐17*, respectively 26, 27. We also searched the literature and found that miR‐17‐5p is more common and significant in GC than is miR‐17‐3p. Thus, we finally focused on miR‐17‐5p.

**Table 1:** The relevant basic information and clinical data of 12 eligible GSE microarrays. The differences between the 3p and 5p arms of miRNAs were ignored when identified the DEMs in GSE datasets on account of the names of miRNAs in TCGA database

Study	Contributors, year	Country	Platform	Gastric cancer (*n*)	Healthy control (*n*)	MicroRNAs involved in study (*n*)	Sample type
GSE93415	Sierzega M *et al*., 2017	Poland	GPL19071	20	20	884	Tissue
GSE78775	Yu B *et al*., 2016	China	GPL10850	28	28	851	Tissue
GSE63121	Zhang X *et al*., 2014	China	GPL8786	15	15	847	Tissue
GSE54397	Chang H *et al*., 2014	South Korea	GPL15159	16	16	1205	Tissue
GSE26595	Lee JS *et al*. 2013	South Korea	GPL8179	60	8	359	Tissue
GSE33743	Carvalho J *et al*. 2012	Portugal	GPL14895	37	4	702	Tissue
GSE30070	Kim CH *et al*. 2011	USA	GPL13742	90	34	414	Tissue
GSE28770	Ahringer J *et al*. 2011	USA	GPL9269	22	22	470	Tissue
GSE85589	Lee J *et al*., 2016	South Korea	GPL19117	7	19	825	Serum
GSE59856	Kojima M *et al*., 2015	Japan	GPL18941	50	150	2555	Serum
GSE61741	Keller A, 2014	Germany	GPL9040	13	94	848	blood
GSE31568	Keller A *et al*., 2011	Germany	GPL9040	13	70	863	blood

**Table 2:** 5p and 3p arms of hsa‐miR‐17 in the top 500 microRNAs according to SNR, as calculated in morpheus. Hsa‐miR‐17‐5p is also known as hsa‐miR‐17 and Hsa‐miR‐17‐3p is also known as hsa‐miR‐17*

Study	Differentially expressed	
	hsa‐miR‐17‐5p	hsa‐miR‐17‐3p
GSE93415	Yes	Yes
GSE78775	Yes	Yes
GSE63121	Yes	No
GSE54397	Yes	Yes
GSE26595	Yes	Yes
GSE33743	Yes	Yes
GSE30070	Yes	No
GSE28770	Yes	No
GSE85589	Yes	Yes
GSE59856	Yes	No
GSE61741	Yes	Yes
GSE31568	No	Yes

**Figure 3:**
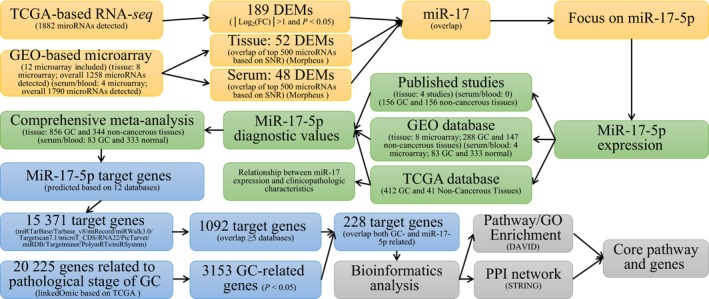
General flowchart. The present study is composed of four procedures performed sequentially: the identification of GC‐related DEMs based on TCGA and GEO datasets, the verification of clinical values based on comprehensive meta‐analysis, the prediction of target genes, and multiple bioinformatics analyses.

The general flowchart is shown in Fig. 3. The present study is composed of four procedures performed sequentially, that is, the identification of GC‐related DEMs based on TCGA and GEO, the verification of clinical values base on comprehensive meta‐analysis, the prediction of target genes, and multiple bioinformatics analyses.

### miR‐17 expression in GC in TCGA database

**Figure 4:**
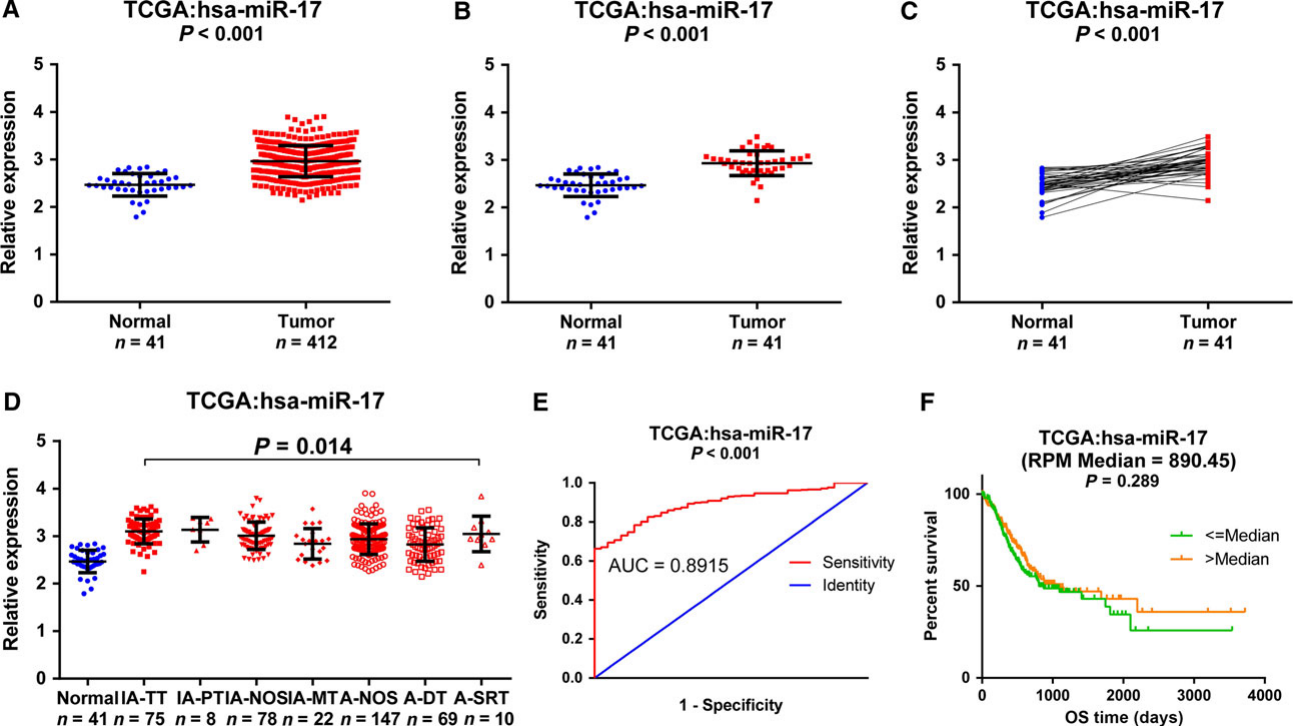
MiR‐17 expression in GC in TCGA database. The expression of miR‐17 in TCGA data was normalized using the logarithm. (A) Comparison of miR‐17 levels in 412 GC tissues and 41 normal gastric mucosal tissues in TCGA data. (B,C) Comparison of miR‐17 levels in 41 pairs of GC tissues and adjacent non‐tumor tissues. (D) Comparison of miR‐17 levels in different histological types of GC tissues. (E) The ROC curve of miR‐17 expression based on TCGA data (AUC = 0.857, 95% CI: 0.808–0.905, *P* < 0.001). (F) Kaplan–Meier survival curves of miR‐17. A‐DT, adenocarcinoma diffuse type; A‐NOS, adenocarcinoma not otherwise specified; A‐SRT, adenocarcinoma signet ring Type; AUC, area under the curve; IA‐MT, intestinal adenocarcinoma mucinous type; IA‐NOS, intestinal adenocarcinoma not otherwise specified; IA‐PT, intestinal adenocarcinoma papillary type; IA‐TT, intestinal adenocarcinoma tubular type; OS, overall survival.

The expression level of miR‐17 was higher in the 412 GC tissues of different pathological stages than in the 41 normal gastric mucosal tissues (*P* < 0.001) (Fig. 4A). The expression level results of miR‐17 for the 41 matched gastric cancer tissues are the same (Fig. 4B,C). The expression of miR‐17 in TCGA data was normalized using the logarithm. Furthermore, we explored the relationship between miR‐17 expression level and clinicopathological characteristics, with the results summarized in Table 3. No significant differences were observed among American Joint Committee on Cancer T, N, M stages, age, and gender. Among GC types, miR‐17 expression level was increased in the tubular and papillary types of intestinal adenocarcinoma and reduced in the diffuse type of adenocarcinoma (*P* = 0.014; Fig. 4D and Table 3). The *P*‐value of the diagnostic power in the receiver operating characteristic (ROC) curve was <0.001 (area under the curve (AUC) = 0.857, 95% CI: 0.808–0.905, *P* < 0.001; Fig. 4E). Additionally, there were no significant differences based on survival analyses (hazard ratio = 1.186, 95% CI: 0.866–1.624, *P* = 0.289) (Fig. 4F).

**Table 3:** Relationship between the expression of miR‐17 and clinicopathological features in GC from TCGA. One‐way analysis of variance and Student's paired or unpaired *t*‐test were used. A‐DT, adenocarcinoma diffuse type; AJCC, American Joint Committee on Cancer; A‐NOS, adenocarcinoma not otherwise specified; A‐SRT, adenocarcinoma signet ring Type; IA‐MT, intestinal adenocarcinoma mucinous type; IA‐NOS, intestinal adenocarcinoma not otherwise specified; IA‐PT, intestinal adenocarcinoma papillary type; IA‐TT, intestinal adenocarcinoma tubular type

Clinicopathological feature		*n*	hsa‐miR‐17 expression (RPM)			
			Mean	SD	*t*/*F*	*P*‐value
Tissue (unmatched)	Normal	41	333.692	159.356	−5.211	<0.001
	Tumor	412	1224.398	1092.287		
Tissue (matched)	Normal	41	333.692	159.356	−6.591	<0.001
	Tumor	41	1001.995	602.321		
Age (years)	<60	124	1086.228	999.872	−1.715	0.087
	≥60	283	1288.305	1132.681		
Gender	Male	266	1254.781	1148.808	0.762	0.447
	Female	146	1169.042	982.366		
Histological type	IA‐TT	75	1489.868	889.374	2.695	0.014
	IA‐PT	8	1559.301	723.730		
	IA‐NO	78	1292.949	1076.012		
	IA‐MT	22	913.999	814.966		
	A‐NOS	147	1165.330	1137.682		
	A‐DT	69	918.369	770.191		
	A‐SRT	10	1626.048	1917.403		
Pathological stage	I	58	1357.578	1038.064	1.063	0.362
	II	128	1122.297	835.139		
	III	183	1285.011	1259.853		
	IV	43	1090.728	1065.495		
AJCC pathological T	T1	21	1192.815	531.405	0.473	0.701
	T2	87	1193.633	1021.903		
	T3	188	1179.104	865.113		
	T4	116	1326.595	1483.968		
AJCC pathological N	N0	129	1232.097	932.042	0.409	0.802
	N1	109	1225.825	1224.321		
	N2	82	1153.218	806.988		
	N3	86	1243.926	1343.528		
	Nx	6	1725.801	1351.577		
AJCC pathological M	M0	364	1221.083	1037.290	0.259	0.772
	M1	29	1160.069	1267.692		
	Mx	19	1386.078	1734.700		

### miR‐17‐5p expression in GC based on the GEO database

**Figure 5:**
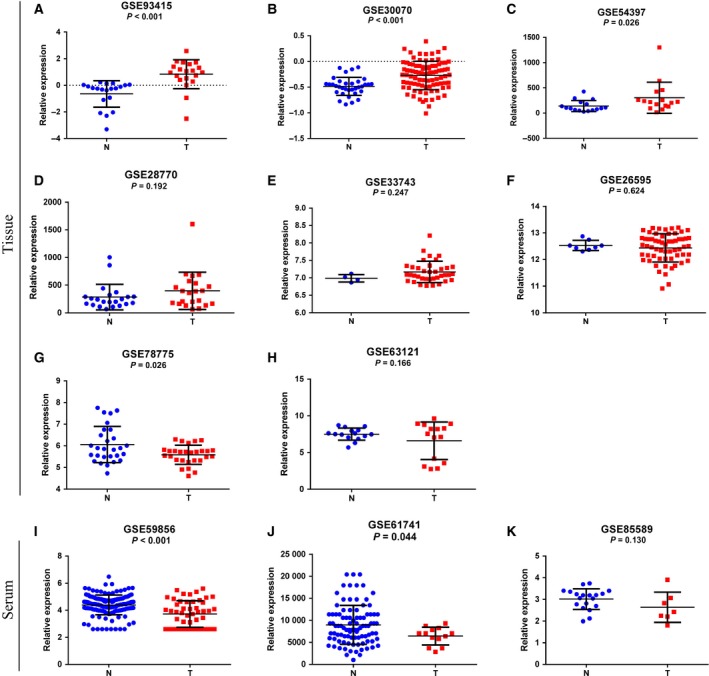
MiR‐17‐5p expression in GC based on the GEO database. (A–H) Expression levels of miR‐17‐5p in GC tissues from the GEO database. (I–K) Expression levels of miR‐17‐5p in GC serum or blood from the GEO database. N, normal gastric mucosal tissues or serum/blood from healthy volunteers; T, tumor tissues or serum/blood from GC patients.

A total of 12 GSE datasets, which consisted of 371 GC samples and 480 healthy control samples, were included in the present study (Table 1). Except for GSE31568, the other 11 GEO datasets all contained miR‐17‐5p (Table 2). Interestingly, the expression level of miR‐17‐5p showed different trends in tissues and serum/blood. The expression of miR‐17‐5p was upregulated in five of the eight GEO datasets in which the sample type was tissue (Fig. 5A–H) but downregulated in all three GEO datasets in which the sample type was serum/blood (Fig. 5I–K).

### Meta‐analysis of miR‐17‐5p expression in GC

**Figure 6:**
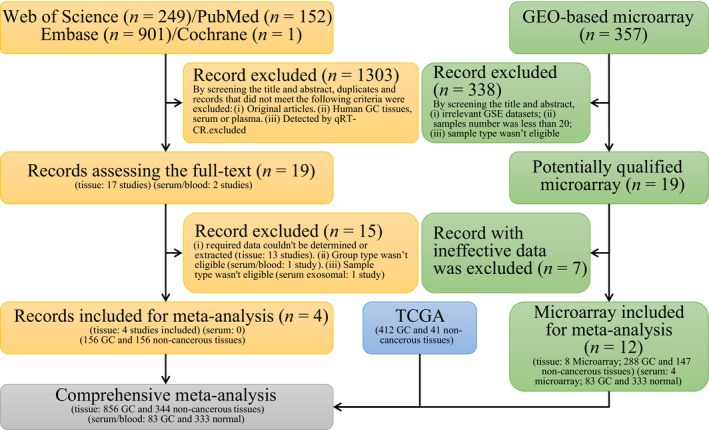
Meta‐analysis flowchart.

**Figure 7:**
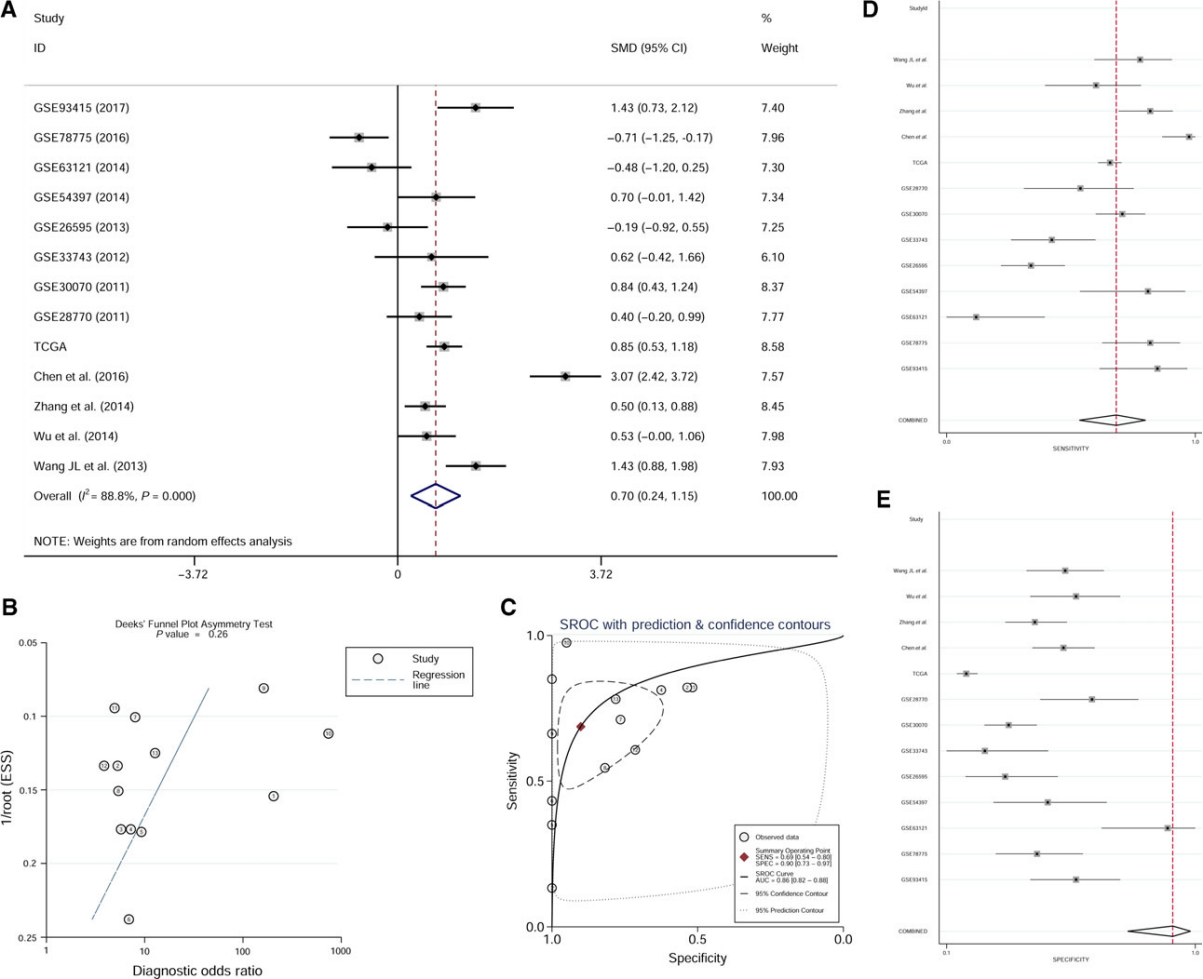
Meta‐analysis of all of the available data of miR‐17‐5p expression level in GC tissues. (A) Forest plot of the meta‐analysis of miR‐17‐5p expression in GC tissues (SMD = 0.695, 95% CI: 0.241–1.150, *P* = 0.003). (B) Funnel plot of the meta‐analysis of miR‐17‐5p expression in GC tissues (Deeks's test: *P* = 0.257). (C) SROC curves for miR‐17‐5p in the diagnosis of GC (AUC = 0.86, 95% CI: 0.82–0.88). (D,E) Forest plot of sensitivity and specificity of miR‐17‐5p (pooled sensitivity = 0.69, 95% CI: 0.54–0.80; pooled specificity = 0.90, 95%CI: 0.73–0.97).

The flowchart for the meta‐analysis is shown in Fig. 6. Regarding the difference in the expression of miR‐17‐5p between GC tissues and adjacent normal gastric mucosal tissues, in addition to the eight GEO datasets (GSE93415, GSE78775, GSE63121, GSE54397, GSE26595, GSE33743, GSE30070, and GSE28770) in which the sample type analyzed was tissue and TCGA database, four additional published studies were included in the meta‐analysis 28, 29, 30, 31. The available data extracted from the original study that were utilized for the meta‐analysis are shown in Table 4. We determined the pooled SMD of miR‐17‐5p to be 0.695 (95% CI: 0.241–1.150, *P* = 0.003; Fig. 7A) using a random‐effects model. The *P*‐value of the heterogeneity test was <0.001 (*I* ^2^ = 88.8%). No obvious publication bias was observed (Deeks's test: *P* = 0.257; Fig. 7B). The diagnostic accuracy was evaluated by plotting an SROC and calculating the AUC (AUC = 0.86, 95% CI: 0.82–0.88; Fig. 7C). The pooled sensitivity was 0.69 (95% CI: 0.54–0.80), and the pooled specificity was 0.90 (95% CI: 0.73–0.97). The forest plot of sensitivity and specificity is presented in Fig. 7D,E.

**Table 4:** Summary of the included studies in the meta‐analysis of miR‐17‐5p expression in GC patient tissues and serum/blood

Study	GC (*n*)	hsa‐miR‐17 expression		HC (*n*)	hsa‐miR‐17 expression		Sample type	Reference
		Mean	SD		Mean	SD		
GSE93415	20	0.834	1.089	20	−0.656	0.998	Tissue	
GSE78775	28	5.590	0.441	28	6.062	0.835	Tissue	
GSE63121	15	6.606	2.544	15	7.507	0.819	Tissue	
GSE54397	16	303.974	308.340	16	140.914	110.512	Tissue	
GSE26595	60	12.437	0.531	8	12.531	0.190	Tissue	
GSE33743	37	7.169	0.306	4	6.986	0.108	Tissue	
GSE30070	90	−0.272	0.278	34	−0.4851	0.178	Tissue	
GSE28770	22	399.372	336.723	22	285.519	229.129	Tissue	
TCGA	412	1224.398	1092.287	41	333.692	159.356	Tissue	
Chen *et al*.	40	1.794	0.326	40	0.916	0.239	Tissue	31
Zhang *et al*.	56	2.427	1.664	56	1.605	1.601	Tissue	30
Wu *et al*.	28	3.595	2.699	28	2.273	2.270	Tissue	29
Wang JL *et al*.	32	1.349	0.489	32	0.649	0.489	Tissue	28
GSE85589	7	2.640	0.696	19	3.014	0.477	Serum	
GSE59856	50	3.722	0.982	150	4.393	0.730	Serum	
GSE61741	13	6442.375	2017.992	94	8987.255	4418.810	Blood	

**Figure 8:**
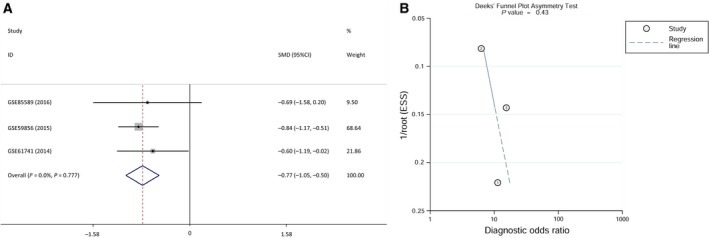
Meta‐analysis of all of the available data for miR‐17‐5p expression levels in GC serum/blood. (A) Forest plot of the meta‐analysis of miR‐17‐5p expression in GC serum/blood (SMD = −0.744, 95% CI: −1.048 to −0.5, *P* < 0.001). (B) Funnel plot of the meta‐analysis of miR‐17‐5p expression in GC serum/blood (Deeks's test: *P* = 0.43).

Next, we also conducted a meta‐analysis on the expression level of miR‐17‐5p in serum/blood in GC. Because there were no eligible studies and relevant TCGA data, only three GEO datasets (GSE59856, GSE61741, and GSE85589) in which the sample type was serum/blood were included in this meta‐analysis (Table 4). The pooled SMD of miR‐17‐5p was −0.774 (95% CI: −1.048 to −0.5, *P* < 0.001; Fig. 8A) using a fixed‐effects model. The *P*‐value of the heterogeneity test was 0.777 (*I* ^2^ = 0%). Additionally, the publication bias was not statistically significant (Deeks's test: *P* = 0.43; Fig. 8B).

### Identification of miR‐17‐5p target genes and bioinformatics analysis

**Figure 9:**
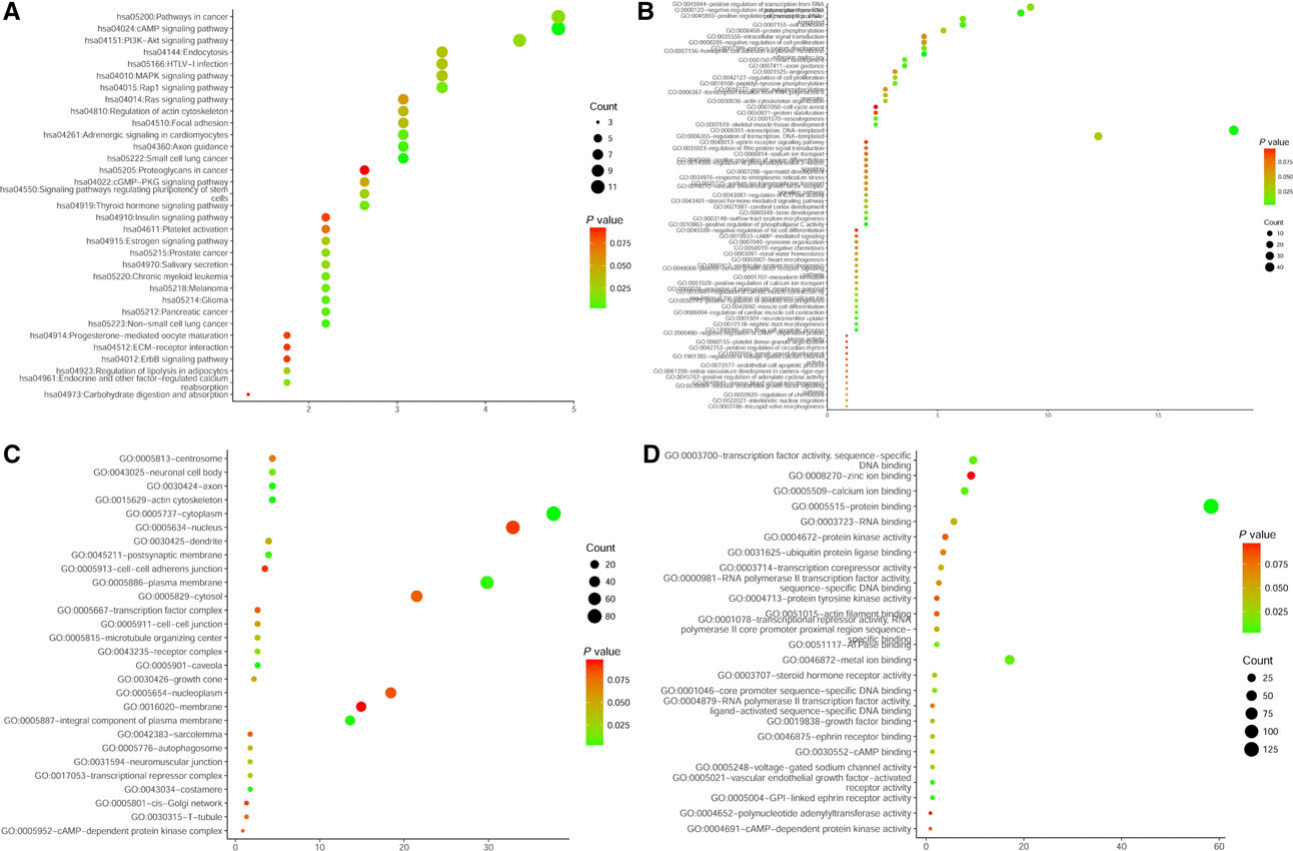
Enriched KEGG pathways and Gene Ontology items of 288 overlapping target genes of miR‐17‐5p. (A) Enriched KEGG pathways. (B) Enriched biological processes. (C) Enriched cellular components. (D) Enriched molecular functions.

**Figure 10:**
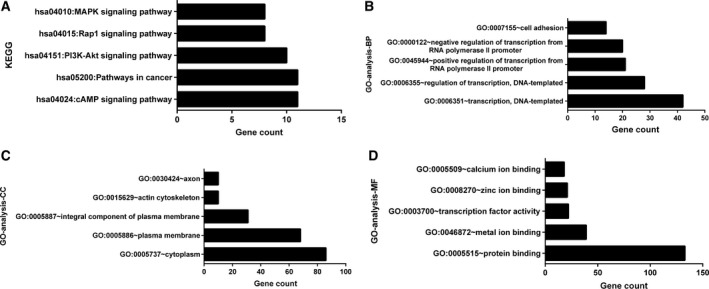
Top five KEGG pathways and GO analysis items. (A) KEGG pathways. (B) Biological processes (BP). (C) Cellular components (CC). (D) Molecular functions (MF).

**Figure 11:**
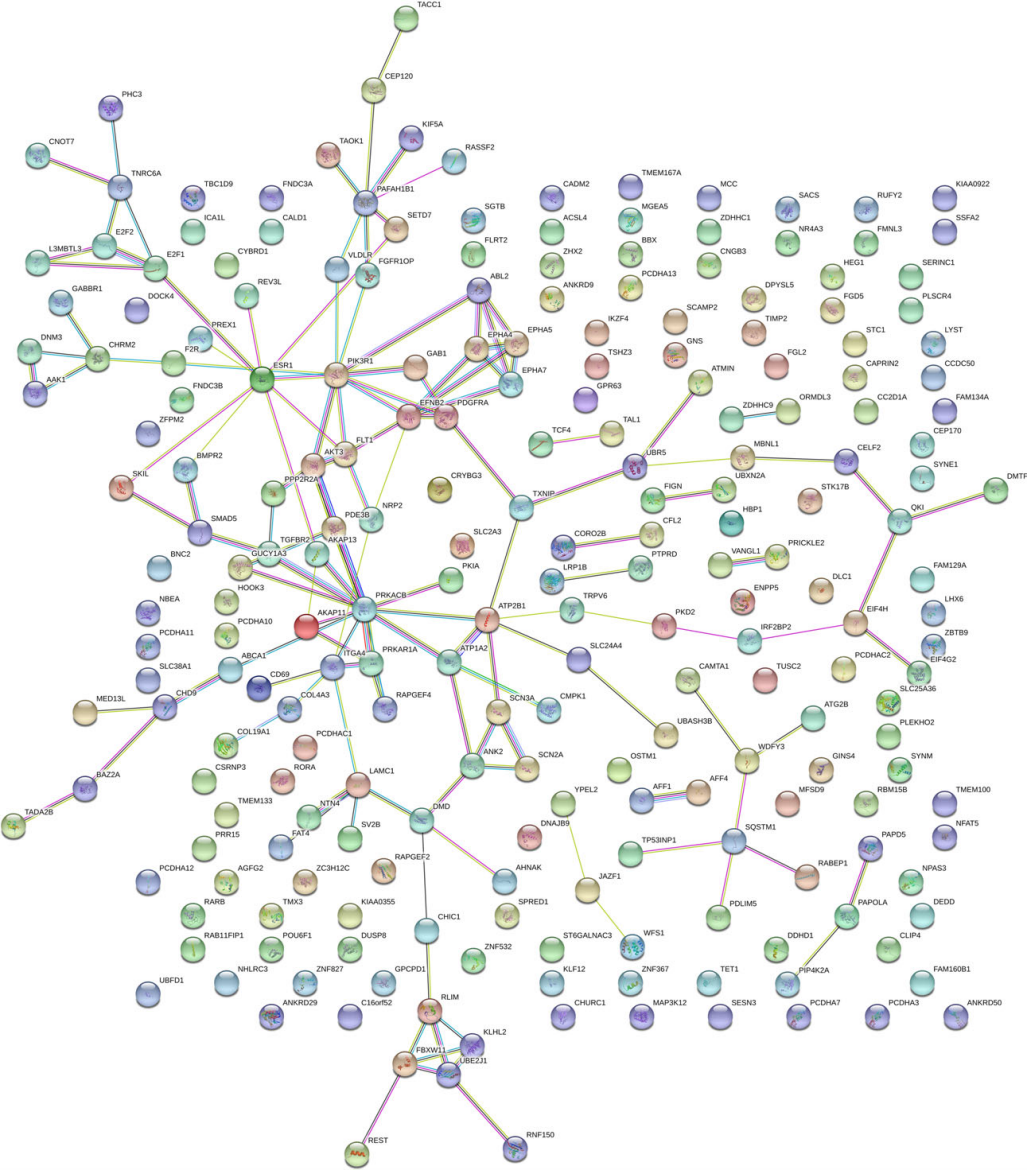
Protein–protein interaction networks of the 288 overlapped target genes of miR‐17‐5p.

**Figure 12:**
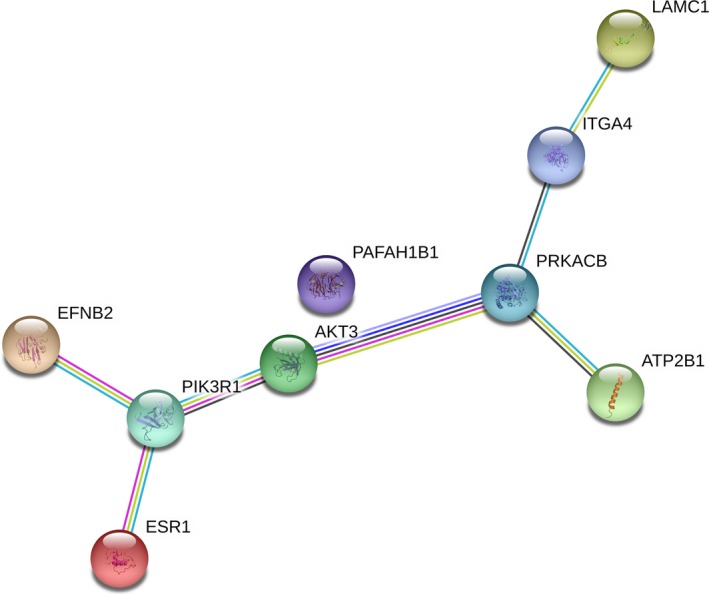
Top nine hub target genes of miR‐17‐5p identified by the PPI network.

Based on 12 target gene prediction databases and TCGA database, 288 prospective target genes of miR‐17‐5p were included (Fig. 3). According to the KEGG pathway analysis and GO enrichment in DAVID, a total of 33 KEGG pathways, 69 GO terms of biological processes, 28 GO terms of cellular components, and 25 GO terms of molecular function were identified (Fig. 9A–D). The top five KEGG pathway and GO terms are listed in Fig. 10. According to the results of our study, 288 genes were highly concentrated in the cAMP, phosphoinositide‐3‐kinase (PI3K)–Akt, Rap1, mitogen‐activated protein kinase (MAPK) signaling pathways and in pathways involved in cancer (*P* < 0.05, Fig. 10A and Table S1). In the GO enrichment, the genes were most likely involved in the biological processes of DNA‐templated transcription, the regulation of DNA‐templated transcription, the regulation of transcription from RNA polymerase II promoter and cell adhesion (*P* < 0.05, Fig. 10B and Table S2), in the cellular components of cytoplasm, plasma membrane, integral component of plasma membrane, actin cytoskeleton, and axon (*P* < 0.05, Fig. 10C and Table S3), in the molecular functions of protein binding, metal ion binding, transcription factor activity, sequence‐specific DNA binding, zinc binding, and calcium binding (*P* < 0.05, Fig. 10D and Table S4). The protein–protein interaction (PPI) networks of the 288 target genes are shown in Fig. 11. The top nine hub target genes of miR‐17‐5p with at least five connections in the PPI network were *PRKACB, ITGA4, PAFAH1B1, PIK3R1, ESR1, EFNB2, ATP2B1, AKT3, and LAMC1* (Fig. 12).

## Discussion

GC, a fatal disease, has attracted increasing attention from clinicians worldwide because of its high morbidity and mortality rates. Despite advancements in life science and medicine, the achievements in diagnosis, treatment and understanding of the pathology do not yet satisfy the needs of patients for earlier diagnosis and longer survival time. In the present study, we integrated the information from next‐generation sequencing (TCGA) analysis and noncoding RNA profiling by microarray (GEO) in GC. As a promising miRNA candidate, miR‐17‐5p appeared in almost all of the datasets, suggesting that there may be some vital connection between miR‐17‐5p and GC.

miR‐17, as one of members of the miR‐17‐92 cluster, is located in an intron of nonprotein coding gene *miR17HG* (the miR‐17‐92 cluster host gene) on chromosome 13 in the human genome 32. The miR‐17‐92 cluster, also termed onco‐miR‐1, is upregulated in several types of cancer, such as lung, breast, stomach, prostate, colon, and pancreatic cancers 33. The other members of the miR‐17‐92 cluster (miR‐18a, miR‐19a, miR‐20a, miR‐19b‐1, and miR‐92a) were not present in all of the GEO and TCGA databases except miR‐17 in our study. As reported, upregulated miR‐17‐5p expression levels enhanced pancreatic cancer proliferation by altering cell cycle profiles 34. Low levels of miR‐17 and miR‐20a as a result of single nucleotide polymorphisms at the promoter of the miR‐17‐92 cluster may decrease the risk of colorectal cancer 35. In patients with recurrent breast cancer, miR‐17‐5p is upregulated in tumor tissues and significantly downregulated in serum as one of the exosomal miRNAs 36. In contrast, statistically significant reductions in the levels of miR‐17 and miR‐19a in plasma have been observed between early and advanced stages of breast cancer 37.

With regard to the expression of miR‐17‐5p in GC, the majority of studies considered that this miRNA is upregulated in GC tissues 28, 29, 30, 31, 38, 39, 40. Consistent with those previous studies, the meta‐analysis in our study revealed that miR‐17‐5p was significantly upregulated in GC tumors. A meta‐analysis of the miR‐17‐92 cluster in various cancers, including GC, indicated a poor prognosis in patients with high expression of this cluster 41. Another meta‐analysis identified 23 significantly upregulated miRNAs, including miR‐17, that were correlated with poor prognosis in gastrointestinal cancers 42. However, neither of the meta‐analyses evaluated the expression of miR‐17‐5p separately in GC. In addition, the expression level of miR‐17‐5p in serum/blood in GC remained controversial. The microarray data in the GSE85589, GSE59856, and GSE61741 datasets suggested lower miR‐17‐5p levels in the serum/blood of GC patients than in healthy control individuals. In a study by Zeng *et al*., the serum levels of miR‐17 were significantly reduced in both GC (*n* = 40) and benign gastric disease (gastric ulcer and gastric polyp) (*n* = 32) patients compared with healthy control individuals (*n* = 36) 43. However, Zhou *et al*., by analyzing mononuclear cells collected from peripheral blood containing circulating tumor cells, concluded that miR‐17 and miR‐106a levels were significantly higher in preoperative (*n* = 41) and postoperative (*n* = 49) GC patients than in healthy volunteers (*n* = 27) 44. Wang *et al*. identified four miRNAs, including miR‐17‐5p, in serum‐circulating exosomes from a cohort of 20 healthy control individuals and 20 GC patients; however, according to this study, the upregulated expression of miR‐17‐5p had no statistical significance 45. Furthermore, Tsujiura *et al*. demonstrated that the plasma concentration of miR‐17‐5p without contamination by cellular nucleic acids was significantly higher in GC patients (*n* = 69) than in healthy controls (*n* = 30) 46. According to the results of our two independent meta‐analyses, miR‐17‐5p expression levels were upregulated in GC tissues but downregulated in the serum/blood of GC patients. Our hypothesis is that GC tumorigenesis may have an effect on the extracellular transport of miR‐10‐5p. However, further research is needed to explore possible mechanisms. Notably, serum/blood samples from 70 GC patients and 263 healthy controls, which came from only three independent studies, were included in the meta‐analysis of miR‐17‐7p expression. Thus, although we conducted a meta‐analysis of miR‐17‐5p expression levels in serum/blood in our study, further investigations are required to confirm the relevant results. However, in GC tissues, the increased expression level of miR‐17‐5p, which was verified in our study, still reveals a promising prospect for miR‐17‐5p as a biomarker in GC. The diagnostic accuracy was evaluated and miR‐17 had increased specificity and sensitivity (Fig. 7C–E).

To investigate the underlying molecular mechanism, we performed a comprehensive bioinformatics analysis. According to 12 miRNA target gene prediction databases and the relevant TCGA data, 288 genes were considered for further analysis. Based on KEGG pathway analysis and GO enrichment, several core pathways and GO terms displayed the potential to play a crucial role in GC. Nine hub target genes, namely *PRKACB, ITGA4, PAFAH1B1, PIK3R1, ESR1, EFNB2, ATP2B1, AKT3,* and *LAMC1,* may have a close association with the tumorigenesis, disease progression, invasion, and metastasis of GC.

Our study may be the first example of the integration of data from the GEO database, TCGA database, and published literature to investigate the possible differential expression of miRNAs and their potential molecular mechanisms in GC. The study identified some core pathways and genes in GC, which may facilitate the further exploration of mechanisms. However, there are some limitations to our study. First, the numbers of miRNAs detected in different GSE chips were different (Table 1), suggesting that some newly discovered miRNAs might be missing. And some miRNAs might be excluded because of our rigorous screening criteria. Second, the meta‐analysis of the expression of miR‐17‐5p in serum/blood of GC needed to be optimized because of insufficiently reliable studies at present. Third, the prediction of target genes was based on different algorithms. More experiments will be needed for validation or even correction and to confirm the KEGG pathway analysis and GO enrichment results.

In conclusion, we believe that miR‐17 may serve as a promising diagnostic marker for GC. miR‐17‐5p promotes the occurrence and development of GC by targeting certain downstream genes. Future studies should be focused on the functions and underlying pathways of miR‐17 in different GC sample types, such as tissue, serum, blood, circulating tumor cells, serum exosomes, and others, to further explore its utility in the diagnosis and molecular therapy of GC.

## Author contributions

WX conceived and designed the project. GFH, QWL, JXY, and LJC performed the experiments. GFH, QWL, JXY, LJC, JD, and KZ analyzed the data. GFH, WX, JD, and KZ wrote the manuscript. All authors read and approved the final manuscript.

## Supporting information


**Table S1.** Pathway enrichment in KEGG databases of the 228 targets of miR‐17‐5p.

**Table S2.** The GO analysis of BP of 228 target genes of miR‐17‐5p.

**Table S3.** The GO analysis of CC of 228 target genes of miR‐17‐5p.

**Table S4.** The GO analysis of MF of 228 target genes of miR‐17‐5p.
