# Supplementary material for: MicroRNA‐17 as a promising diagnostic biomarker of gastric cancer: An investigation combining TCGA, GEO, meta‐analysis, and bioinformatics
Source: FEBS Open Bio. 2018 Aug 30;8(9):1508–23. doi: 10.1002/2211-5463.12496 (PMC6120248; doi:10.1002/2211-5463.12496)
Supplement: Supplementary file 5 [file FEB4-8-1508-s005.docx]

Table S1. Pathway enrichment in KEGG databases of the 228 targets of miR-17-5p.

Table S2. The GO analysis of BP of 228 target genes of miR-17-5p.

Table S3. The GO analysis of CC of 228 target genes of miR-17-5p.

Table S4. The GO analysis of MF of 228 target genes of miR-17-5p.
